# Evaluation of Exotically-Derived Soybean Breeding Lines for Seed Yield, Germination, Damage, and Composition under Dryland Production in the Midsouthern USA

**DOI:** 10.3389/fpls.2017.00176

**Published:** 2017-02-27

**Authors:** Nacer Bellaloui, James R. Smith, Alemu Mengistu, Jeffery D. Ray, Anne M. Gillen

**Affiliations:** ^1^Crop Genetics Research Unit, USDA Agricultural Research ServiceStoneville, MS, USA; ^2^Crop Genetics Research Unit, USDA Agricultural Research ServiceJackson, TN, USA

**Keywords:** soybean nutrition, seed composition, mineral nutrition, seed protein, seed oil, germination, seed diseases

## Abstract

Although the Early Soybean Production System (ESPS) in the Midsouthern USA increased seed yield under irrigated and non-irrigated conditions, heat stress and drought still lead to poor seed quality in heat sensitive soybean cultivars. Our breeding goal was to identify breeding lines that possess high germination, nutritional quality, and yield potential under high heat and dryland production conditions. Our hypothesis was that breeding lines derived from exotic germplasm might possess physiological and genetic traits allowing for higher seed germinability under high heat conditions. In a 2-year field experiment, breeding lines derived from exotic soybean accessions, previously selected for adaptability to the ESPS in maturity groups (MG) III and IV, were grown under non-irrigated conditions. Results showed that three exotic breeding lines had consistently superior germination across 2 years. These lines had a mean germination percentage of >80%. Two (25-1-1-4-1-1 and 34-3-1-2-4-1) out of the three lines with ≥80% germination in both years maintained high seed protein, oleic acid, N, P, K, B, Cu, and Mo in both years. Significant (*P* < 0.05) positive correlations were found between germination and oleic acid and with K and Cu in both years. Significant negative correlations were found between germination and linoleic acid, Ca, and hard seed in both years. There were positive correlations between germination and N, P, B, Mo, and palmitic acid only in 2013. A negative correlation was found between germination and green seed damage and linolenic acid in 2013 only. Seed wrinkling was significantly negatively correlated with germination in 2012 only. A lower content of Ca in the seed of high germinability genotypes may explain the lower rates of hard seed in those lines, which could lead to higher germination. Many of the differences in yield, germination, diseases, and seed composition between years are likely due to heat and rainfall differences between years. The results also showed the potential roles of seed minerals, especially K, Ca, B, Cu, and Mo, in maintaining high seed quality. The knowledge gained from this research will help breeders to select for soybean with high seed nutritional qualities and high germinability.

## Introduction

The development of the Early Soybean Production System (ESPS) in the Midsouthern resulted in higher yield under irrigated and non-irrigated conditions (Heatherly, 1999). However, high heat and drought in the ESPS are still major environmental stress factors, resulting in poor seed quality (Mengistu and Heatherly, 2006; Smith et al., 2008; Mengistu et al., 2009, 2010) and yield reduction for heat sensitive soybeans, especially under dryland conditions. In addition, early-maturing cultivars grown in the Midsouth USA are often exposed to higher temperature, rainfall, and relative humidity, resulting in high seed infection (TeKrony et al., 1980, 1984) from pathogens such as *Phomopsis* spp. (Kmetz et al., 1974, 1978, 1979), reduction of seed quality (low viability, moldy seed, and reduced emergence; Kmetz et al., 1978; TeKrony et al., 1980), and lower market grade and reduced quality of meal and oil (Hepperly and Sinclair, 1978).

Previous research showed that high temperature and high humidity promote the development of seed with substandard germination and poor seed quality due to diseases such as *Phomopsis longicolla* Hobbs (Thomison et al., 1990; Tekrony et al., 1996; Mengistu and Heatherly, 2006), seed coat wrinkling (Franca-Neto et al., 1988); seed coat shriveling (Franca-Neto et al., 1993; Spears et al., 1997), weathering (Keith and Delouche, 1999), and hard seed (impermeable seed coat; Gibson and Mullen, 1996; Spears et al., 1997; Kebede et al., 2014). Identifying soybean lines with heat-tolerance under dryland conditions could be an effective way to further optimize seed yield and maintain high seed quality (viability, germination, vigor, and composition).

Germinability (germination and vigor) is an important trait for seed producers, and seed composition (seed protein, oil, fatty acids, and mineral nutrition) is important for seed consumers. For example, in Mississippi the minimum germination rate required for certified seed is 80%, and seed lots with less than a 60% germination rate are illegal to sell (Keith and Delouche, 1999). High germination is essential for adequate stand establishment and successful crop production. Previous research reported that the ancestors of modern soybean cultivars in the USA lack high germinability (Smith et al., 2008). Without the introgression of new genetic diversity from exotic germplasm into the breeding gene pool used by commercial seed companies, the new cultivars of the future may also lack high germinability. Smith et al. (2008) identified soybean germplasm accessions with high seed germinability for seed produced under high temperature environments in the ESPS of the Midsouthern USA. They reported that 63 accessions were identified as having a mean standard field germination of ≥90% as well as < 10% hard seededness, *P. longicolla* infection and wrinkled seed coat. They were able to identify genotypes with seed traits that can be used in a breeding program to develop cultivars with high seed germinability for use under high temperature production environments such as in ESPS. Salmeron et al. (2014) studied maturity group choices for early and late planting dates under Midsouthern environments using eight locations in 2012 and 10 locations in 2013, four planting dates and 16 cultivars of maturity MG III through VI. They showed that MG IV and V cultivars had higher average yield in early-planting systems, but late MG III to late MG IV cultivars had higher yield in late-planting. It was explained that the main characteristic of the better yielders, for example MG IV cultivars, was that the cultivars were more stable across different environments for early and late planting, and that there was a reduced risk for low yield (Salmeron et al., 2014).

Seed composition is another critical quality trait because soybean seed is a major source of protein and oil (saturated fatty acids such as palmitic and stearic, and unsaturated fatty acids such as oleic, linoleic, and linolenic; Wilson, 2004). Also, soybean seed contains macro- and micro-minerals (Sale and Campbell, 1980; Bellaloui et al., 2015). Comparative studies of soybean seed quality among producing countries showed that US soybeans and soybean meal have lower protein contents than Brazil but higher protein than Argentina (Karr-Lilienthal et al., 2004; Thakur and Hurburgh, 2007), affecting the global competitive market of US soybean. On the other hand, US soybeans had the highest concentration of total essential amino acids, making US soybeans superior in protein quality compared to Brazilian and Chinese cultivars (Grieshop and Fahey, 2001; Karr-Lilienthal et al., 2004; Oltmans-Deardorff et al., 2013). A study of 105 soybean genotypes indicated that US cultivars had on average 41.3% protein and 19.9% oil content on the seed dry mass; however, Japanese and South Korean cultivars contained on average 44.5% protein and 18.1% oil (Shi et al., 2010). This difference is due to different genetic backgrounds (Shi et al., 2010), water availability (Rotundo and Westgate, 2009; Rotundo et al., 2014), temperature (Dornbos and Mullen, 1992; Piper and Boote, 1999; Bellaloui et al., 2009a), and soil fertility (Nakasathien et al., 2000; Ray et al., 2006; Bellaloui et al., 2009b). Therefore, improvement of soybean seed composition for protein and oil content has been critical for almost a decade (Durham, 2003). To address soybean seed composition quality, the United Soybean Board initiated the Better Bean Initiative (BBI) and its Technology Utilization Center (TUC) to improve soybean composition and to keep U.S. soybeans competitive in the world market.

One of the goals of the Better Bean Initiative, launched in 2000, was to modify the ratios of fatty acids (high oleic and low linolenic) in oil processing because high oleic and low linolenic acids contribute to the oxidative stability of the oil and improved shelf-life. It was suggested that the most desirable phenotype for soybean oil is <7% saturates (palmitic and stearic acids), >55% oleic acid, and <3% linolenic acid (Lee et al., 2009). These oils would have multiple uses as edible and processed oils (Wilson, 2004). Therefore, it is useful and critical to soybean breeders to have information on the fatty acid composition of new soybean lines.

To date, little has been done on developing high heat tolerant soybean genotypes with high seed quality characteristics under high heat, high humidity, and drought environments such as in the ESPS in the Midsouthern USA. Therefore, the objective of the current research was to evaluate previously developed breeding lines derived from exotic germplasm for yield, geminability, and seed nutritional value under the production environment of the ESPS without relying on irrigation. Further, we wanted to investigate possible physiological, genotypic, and environmental factors contributing to high germinability under dryland production.

## Materials and methods

### Description of experimental breeding lines

The seven breeding lines and nine check cultivars evaluated in this study are shown in Table 1. Breeding lines 04025-41, 25-1-1-4-1-1, and 34-3-1-2-4-1 were derived from PI 587982A, which was identified by Smith et al. (2008) to have high germinability. The other parent for each of the above three lines was DT98-9102, DT98-9102, and DT97-4290 (Paris et al., 2006), respectively. Breeding line 24-2-1-2-1-2 was derived from DT98-9102 × PI 603756. The latter PI was also identified by Smith et al. (2008) as having high germinability. Each of the above breeding lines is considered to have 50% exotic parentage. Breeding lines LG03-4561-14 and LG03-4561-19 are sister lines from the same F_2_ plant developed by R.L. Nelson and adaptively selected by J.R. Smith for the ESPS. These two lines have 19% exotic parentage derived from PIs 68508 and 445837. Their immediate parents are LG99-5106 and LG97-9226. Breeding line LG04-1459-6 is derived from S32-Z3 × LG00-3056, but has 25% exotic parentage from PIs 361064, 407710, 189930, and 68600. Two public cultivars from Illinois were included in the study; Dwight (Nickell et al., 1998) and LD00-3309 (Diers et al., 2006). Five commercial cultivars (AG3803, AG3905, AG4403, AG4903, and AG5606) developed by the Monsanto Corporation were included, along with one cultivar developed by Hornbeck Seed Company (C4926) and one cultivar developed by Delta King seed company (DK4866). The genotypes used here were categorized into three groups: (1) breeding lines derived from exotic parental accessions and previously identified to have high germinability under irrigation in the ESPS (04025-41, 25-1-1-4-1-1, 34-3-1-2-4-1, and 24-2-1-2-1-2; all 50% exotic); (2) cultivars (checks); and (3) breeding lines derived from exotic parental accessions and previously identified to have high yield potential under irrigation in the ESPS [(LG03-4561-14 and LG03-4561-19, 19% exotic); (LG04-1459-6, 25% exotic)].

**Table 1:** **The 16 soybean genotypes grown under dryland conditions in a 2-year field experiment**.

**Genotype**	**Maturity group**	**Flowering date**	**R8**		**Germination**		
MS exotic breeding lines^a^		2012	2013	2012	2013	2012	2013
04025-41	IV	Jun 12	Jun 29	Sep 6	Sep 13	57.3	81.7
24-2-1-2-1-2	IV	Jun 6	Jun 22	Sep 1	Sep 6	70.7	91.3
25-1-1-4-1-1	IV	Jun 9	Jun 29	Sep 10	Sep 15	87.3	94.3
34-3-1-2-4-1	IV	May 30	Jun 22	Aug 22	Aug 27	92.0	96.0
**CULTIVARS**							
AG3803	III	May 20	Jun 5	Aug 9	Aug 24	36.3	50.3
AG3905	III	May 20	Jun 4	Aug 13	Aug 24	55.0	78.7
AG4403	IV	May 20	Jun 9	Aug 15	Aug 29	70.7	78.0
AG4903	IV	May 20	Jun 8	Aug 27	Sep 12	43.0	51.7
AG5606	V	Jun 6	Jun 22	Sep 15	Sep 26	47.3	47.3
C4926	IV	May 31	Jun 22	Sep 5	Sep 13	41.7	54.7
DK4866	IV	May 20	Jun 5	Aug 26	Sep 7	34.0	41.0
Dwight	II	May 18	Jun 3	Aug 9	Aug 20	49.0	58.7
LD00-3309	IV	May 20	Jun 7	Aug 16	Aug 21	51.0	44.3
**IL EXOTIC BREEDING LINES**^b^							
LG03-4561-14	III	May 20	Jun 7	Aug 7	Aug 21	60.3	83.0
LG03-4561-19	III	May 20	Jun 4	Aug 2	Aug 17	65.0	70.0
LG04-1459-6	IV	May 20	Jun 17	Aug 14	Aug 27	80.0	81.7
LSD		1.11	0.57	1.25	1.88	5.1	6.1

a MS breeding lines, Mississippi breeding lines;

b *IL breeding lines, Illinois breeding lines; R8, full maturity stage. The experiment was conducted in 2012 and 2013 at the Jamie Whitten Delta States Research Center, Stoneville, MS*.

### Field management and growth conditions

The experiments were machine planted on 13 April 2012 and 30 April 2013 at the Jamie Whitten Delta States Research Center in Stoneville, MS, USA. The experimental design in each year was a randomized complete block design with three replications. Experimental units were 4-row plots with a 0.91 m row spacing. Plots were 5.79 m long at planting, but end trimmed to 4.88 m long after R1 (beginning bloom; all reproductive stages according to Fehr and Caviness, 1977) and before R6 (full seed-fill). The middle two rows of each plot were harvested with a small plot combine shortly after R8 (full maturity) and weighed as an estimate of seed yield based on 9% moisture. All estimates of seed characteristics (composition, damage, disease, and germinability) were made on this seed for each plot. A field design was implemented so that all plots were accessible for direct combine harvest whenever they were ready for harvest. As such, even though the maturity groups (MG) ranged from II to V, all plots were timely harvested by the combine as they matured. That is to say, the experiment was not harvested as a group of plots after the last plot matured, but rather over an extended period shortly after each plot arrived at harvest maturity. Timely harvest of each plot was implemented to reduce any potential effect of seed weathering bias. All plots were grown under dry land conditions, with no supplemental irrigation to relieve any drought stress.

Beginning bloom (R1) and full maturity (R8) were recorded for each plot. After R8 and before harvest, plant height and lodging were recorded. Size of harvested seed was estimated as g per 100 seed.

### Soil minerals, N, S, and C analysis

Nutrients in the soil were analyzed at the University of Georgia's Soil, Plant, and Water Laboratory in Athens, GA. Concentrations of minerals were analyzed on a 5 g soil: 20 ml Mehlich-1 solution and the concentrations were determined using inductively coupled plasma spectrometry (Thermo Jarrell-Ash Model 61E ICP and Thermo Jarrell-Ash Autosampler 300). Soil N, S, and C were determined based on the Pregl-Dumas method (Dumas, 1831; Holmes, 1963; Childs and Henner, 1970) using a C/N/S elemental analyzer having thermal conductivity cells (LECOCNS-2000 elemental analyzer, LECO Corporation, St. Joseph, MI, USA). Briefly, a 0.25 g sample of soil was combusted in an oxygen atmosphere at 1350°C, converting elemental N, S, and C into N_2_, SO_2_, and CO_2_. The gases were then passed through infrared cells and N, S, and C were determined by the elemental analyzer. Average composite random soil samples (four random composite samples across the field), taken at the beginning of the vegetative stage, showed no nutrient deficiency in the soil. The soil texture (clay soil) was in percentage (%) sand = 18, silt = 33.6, and clay = 48.4 with C = 1.4%, N = 0.14%, and organic matter = 1.9%. Nutrient contents were (mg/kg) B = 2.9, Cu = 15.2, Zn = 68.7; and (g/kg) Ca = 5.4, Fe = 21.3, K = 2.7, Mg = 3.5, P = 0.35, and S = 0.21. Leaf analyses did not show any nutrient deficiency (data not shown).

### Seed minerals, N, S, and C analysis

In all seed analyses, dried seed samples were ground to pass through a 1 mm sieve using a Laboratory Mill 3600 (Perten, Springfield, IL, USA) and ground dried samples were used for analysis. The grinding of all samples in this study was performed under room temperature conditions. Nutrient contents in samples were determined by digesting a 0.6 g ground sample in HNO_3_ in a microwave digestion system and nutrients were estimated using inductively coupled plasma spectrometry (Thermo Jarrell-Ash Model 61E ICP and Thermo Jarrell-Ash Autosampler 300; Bellaloui et al., 2011, 2014). Measurements of N, C, and S were conducted on a 0.25 g sample. The samples were combusted, and the percentages of C, N, and S were determined using the C/N/S elemental analyzer (Bellaloui et al., 2011, 2014).

### Seed analysis for protein, oil, and fatty acids

Dried seed samples were ground to pass through a 1 mm sieve using a Laboratory Mill 3600 (Perten, Springfield, IL, USA) as described above. Protein, oil, and fatty acids in mature seeds were analyzed according to the detailed methods reported by Bellaloui et al. (2009a, 2010, 2014). Briefly, a 25 g ground sample was analyzed for protein, oil, and fatty acids by near infrared reflectance (Wilcox and Shibles, 2001; Bellaloui et al., 2009a, 2010) using a diode array feed analyzer AD 7200 (Perten, Springfield, IL, USA). The calibration equation was developed by the University of Minnesota using Perten's Thermo Galactic Grams PLS IQ software using conventional chemical protocols with AOAC methods (AOAC, 1990a,b). Protein and oil were expressed on a dry weight basis (Wilcox and Shibles, 2001; Boydak et al., 2002; Bellaloui et al., 2010, 2014). Fatty acid contents (palmitic, stearic, oleic, linoleic, and linolenic acids) were determined on an oil basis (Bellaloui et al., 2009a, 2014).

### Boron determination

The concentration of boron in seeds were measured using the azomethine-H method described by Lohse (1982) and Dordas et al. (2007). Briefly, seed samples were ground to pass through a 1 mm sieve using a Laboratory Mill 3600 (Perten, Springfield, IL, USA) as described above. A ground sample of 1.0 g was ashed at 500°C, extracted with 20 ml of 2 M HCl at 90°C for 10 min, and then a 2 ml sample of the filtered mixture was added to 4 ml of buffer solution (containing 25% ammonium acetate, 1.5% EDTA, and 12.5% acetic acid). A volume of 4 ml of fresh azomethine-H solution (0.45% azomethine-H and 1% of ascorbic acid; John et al., 1975) was then added. Boron concentration was determined in seeds by a Beckman Coulter DU 800 spectrophotometer (Beckman Coulter, Inc., Brea, CA, USA) at 420 nm (Bellaloui et al., 2014).

### Iron determination

The Fe concentration in seeds was measured according to Bandemer and Schaible (1944) and Loeppert and Inskeep (1996). Seed samples were ground using a Laboratory Mill 3600 (Perten, Springfield, IL, USA) as described above, and samples were then acid digested and extracted, with the resulting reduced ferrous Fe reacting with 1,10-phenanthroline as described by Bellaloui et al. (2011, 2014). Briefly, samples of 2 g of ground sample were acid digested, and the soluble constituents were dissolved in 2 M of HCl. A volume of 4 ml of an aliquot containing 1–20 μg of iron of the sample solution was transferred into a 25 ml volumetric flask and diluted to 5 ml using 0.4 M HCl. A volume of 1 ml of Quinol solution was added to the 5 ml diluted sample solution and mixed. A volume of 3 ml of the phenanthroline solution and 5 ml of the tri-sodium citrate solution (8% w/v) was added. Distilled water was then added to dilute the solution to 25 ml and then incubated at room temperature for 4 h. Phenanthroline reagent solution of 0.25% (w/v) in 25% (v/v) ethanol and quinol solution (1% w/v) was prepared. Concentrations ranging from 0.0 to 4 μg ml^−1^ of Fe in 0.4 M HCl were prepared for the standard curve. The concentration of Fe was determined by a Beckman Coulter DU 800 spectrophotometer at an absorbance of 510 nm.

### Phosphorus determination

The concentration of P in seed was determined by the yellow phosphor-vanado-molybdate complex method according to Cavell (1955). The detailed description of the method was previously reported by Bellaloui et al. (2009a, 2014). Briefly, seed samples were ground using a Laboratory Mill 3600 (Perten, Springfield, IL, USA) as described above, and a sample of 2 g of ground seed samples was ashed at 500°C, and then 10 ml of 6 M HCl were added. The samples were then placed in a water bath at 100°C until the solution evaporated to dryness. After P was extracted with 2 ml of 36% v/v HCl under heat and filtration, 5 ml of 5M HCl and 5 ml of ammonium molybdate–ammonium metavanadate reagent were added to 5 ml of the filtrate. Ammonium molybdate–ammonium metavanadate was prepared by dissolving 25 g of ammonium molybdate and 1.25 g of ammonium metavanadate in 500 ml of distilled water. A standard curve was produced by preparing a standard solution of phosphorus in a range of concentrations from 0 to 50 μg ml^−1^ using dihydrogen orthophosphates. The measurement of P concentration was conducted using a Beckman Coulter DU 800 spectrophotometer at an absorbance of 400 nm.

### Seed germination, seed vigor, hard seed coat, and seed damage evaluations

Seed assays for standard germination and hard seed were conducted on 200 seeds per plot by the State Seed Testing Laboratory, Mississippi State, MS following the protocol of the Association of Official Seed Analysts (2001). An assay for seed vigor (accelerated-aging germination test) was also conducted by the State Seed Laboratory on a 42 g sample of seed from each plot following standard procedures (Association of Official Seed Analysts, 2002).

Visual ratings of seed coat wrinkling were taken as described by Smith et al. (2008). In short, ratings were taken from seed harvested from each plot as the percentage of visibly wrinkled seed coat surfaces per total visible seed coat surface area. The same visual rating system was used to estimate green seed damage [(Federal Grain Inspection Service (FGIS), 2013)]. Damage ratings (Grain inspection handbook Book II Soybean, 2013) were made for total seed damage for each plot by certified grain inspectors at MidSouth Grain Inspection Service (Stoneville, MS) using a random 125 g sample from each plot.

### Fungus identification and evaluation

Twenty-five seeds from each plot were disinfected in 0.25% NaOCl for 60 s, blotted dry, plated on acidified potato dextrose agar (APDA; Difco Laboratories, Detroit, MI), and incubated for 7 days at 24°C. *Cercospora kukuchii* was identifiable when cultured on APDA by purple coloration produced on media and the color of the seed coat whereas *P. longicolla* needed further steps to confirm and validate its identity. Identification of *P. longicolla* was based on a single-monoconidial isolate where 10 individual cultures obtained from a monoconidial isolate were evaluated for cultural characteristics (Hobbs et al., 1985; Kulik and Sinclair, 1999; Mengistu et al., 2009, 2007). Each isolate was examined for sporulation, dimensions of conidia, pattern of stroma, and the presence or absence of beta conidia and perithecia (Hobbs et al., 1985; Barnett and Hunter, 1998; Kulik and Sinclair, 1999).

### Experimental design and statistical analysis

The experimental design was a randomized complete block with three replicates (Rep). Analysis of variance was performed using PROC MIXED in SAS [Statistical Analysis System, Copyright 2002–2010, Cary, NC, USA; Windows Version 6.1.7601 (SAS Institute, 2002–2010)]. Year and genotype were modeled as fixed effects. Rep within year was considered as a random effect. Residuals of the random effect factor were shown as covariance parameters in the tables. The residuals refer to Restricted Maximum Residual Likelihood (REML) values, which reflect the total variance of the random parameters in the model. Means were separated by Fisher's protected LSD (0.05). The level of significance of ≤ 0.05 was used in all measured parameters. Correlation was performed by using Prism (version 6.05) GraphPad Software, La Jolla, CA 92037^1^. The correlation was conducted based on the mean values of measured variables.

## Results

### Weather components

**Figure 1:**
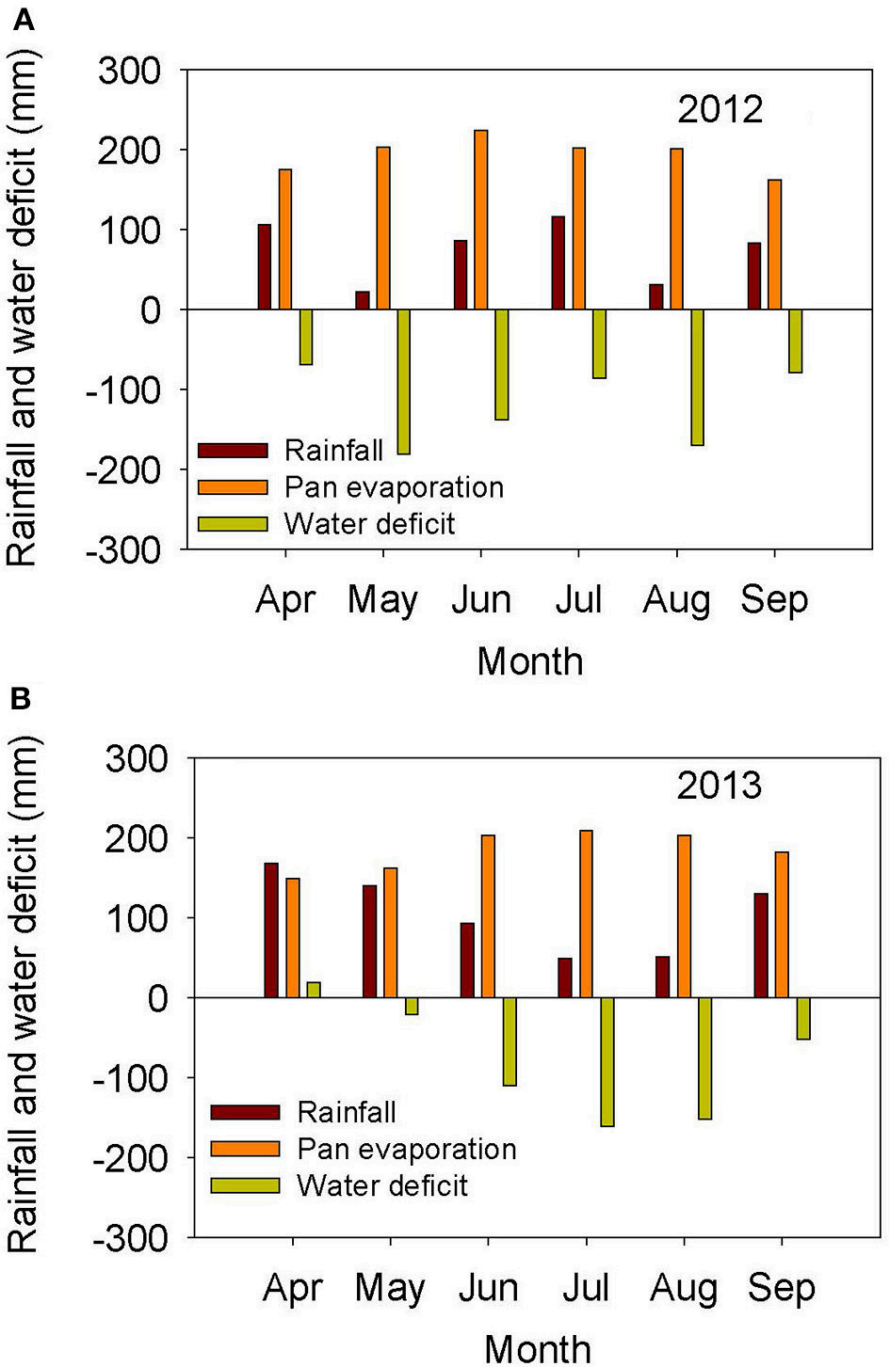
**Rainfall (mm), pan evaporation (mm), and water deficit (mm) during the growing season in 2012 (A)** and 2013 **(B)**. The experiment was conducted in 2012 and 2013 at the Jamie Whitten Delta States Research Center, Stoneville, MS.

**Figure 2:**
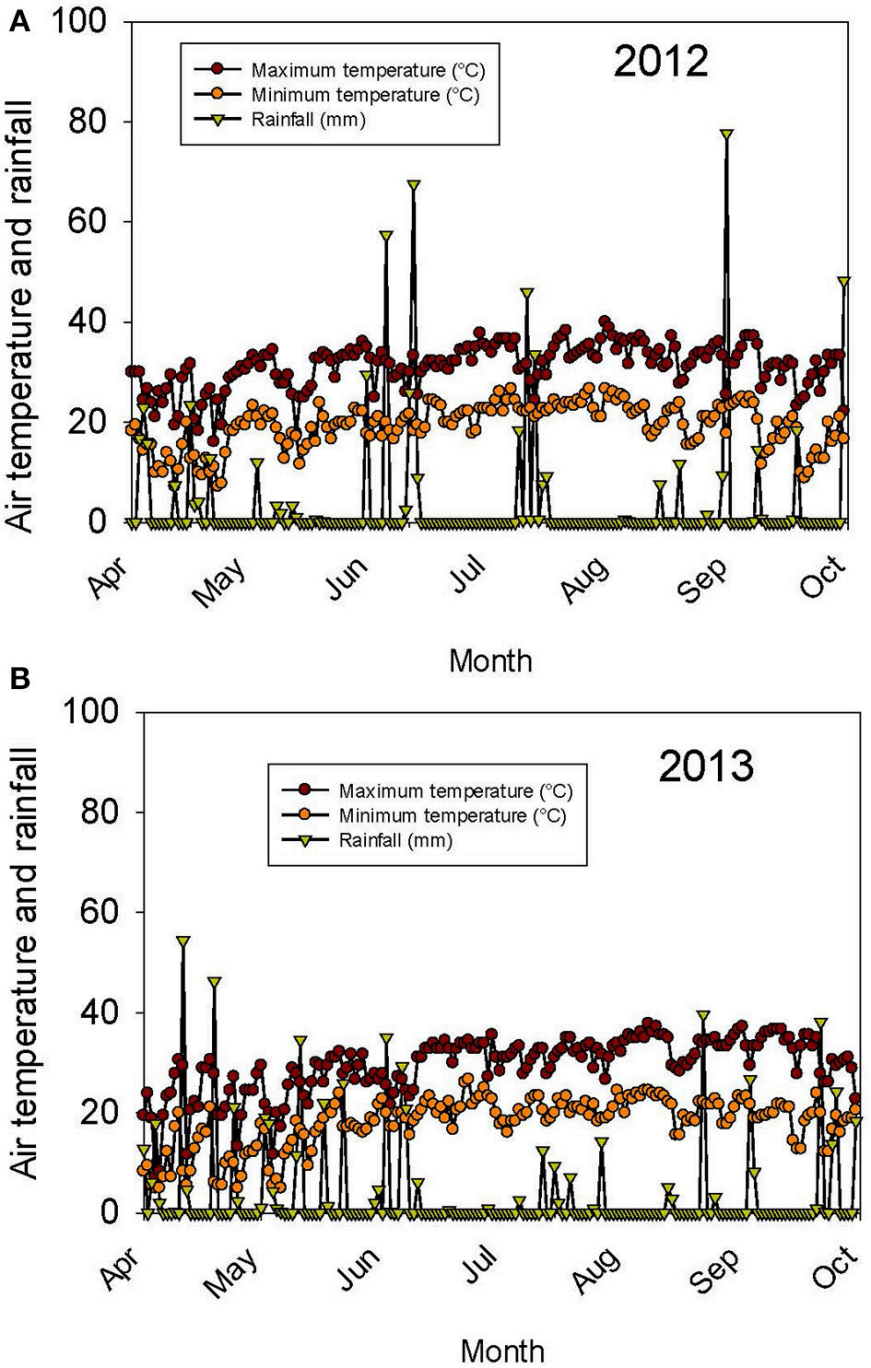
**Continuous pattern of air temperature (°C) and rainfall (mm) during the growing season in 2012 (A)** and 2013 **(B)**. The experiment was conducted in 2012 and 2013 at the Jamie Whitten Delta States Research Center, Stoneville, MS.

**Figure 3:**
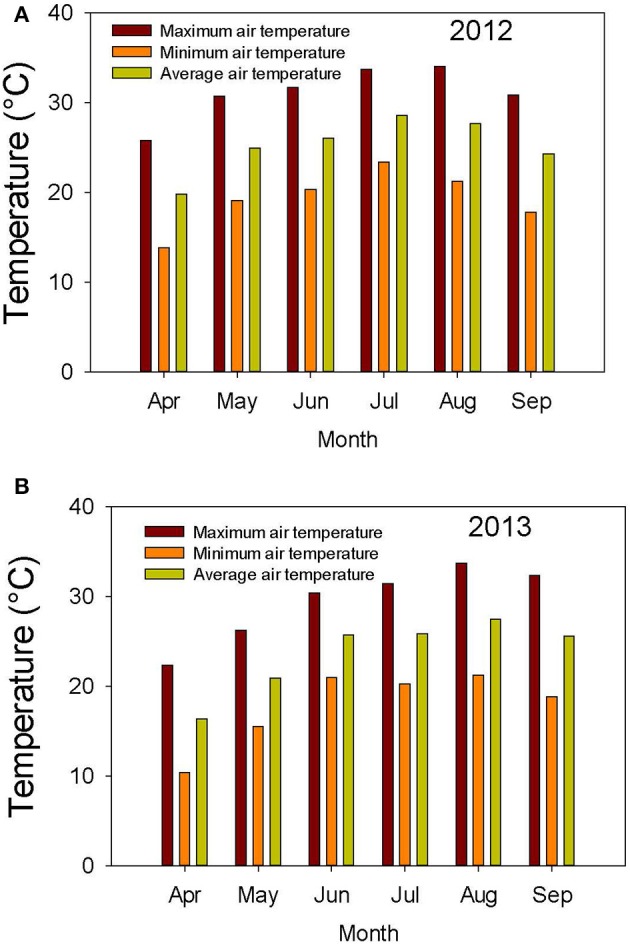
**Air temperature (°C) during the growing season in 2012 (A)** and 2013 **(B)**. The experiment was conducted in 2012 and 2013 at the Jamie Whitten Delta States Research Center, Stoneville, MS.

The weather in the Midsouth is characterized by high heat and drought during the growing season in addition to other abiotic and biotic stress factors such as high humidity and diseases such as charcoal rot and phomopsis seed decay. Based on the weather data for 2012 and 2013 (MSUCares, 2016), the month of July in 2013 showed a higher water deficit than in 2012 (−161 mm in 2013 vs. −86 mm in 2012; Figures 1A,B). The month of July is an important growing period that coincides generally with the seed-fill stage, especially for early-maturing soybean genotypes. Also, the daily rainfall (Figures 2A,B) showed that 2012 received higher rainfall during June and July compared to 2013, but 2013 received more rainfall during August and September than in 2012, indicating differences in rainfall pattern, which may benefit soybean growth and yield for some genotypes as opposed to others due to differences in maturities. The monthly temperature data showed that 2012 was hotter in May through July than in 2013 (Figures 3A,B), although the pattern of temperature during the growing season was different (Figures 2A,B). In addition, R8 dates among genotypes ranged from 44 days in 2012 to 40 days in 2013, potentially exposing different genotypes to different rainfall and temperature environments in each year. The later planting date in 2013, causing all genotypes to mature later that year, created a potential difference with 2012 for the environment in which each genotype matured.

### Analysis of variance

ANOVA showed that year and genotype were the main source of variability (Table 2). Although year × genotype interactions showed significant effects for some seed quality components, the *F*-values were generally low compared with those of main effect factors, except for total seed damage and seed diseases (Table 2). The year × genotype interactions are probably due to yearly changes in rainfall (Figures 1A,B) and heat (Figures 2A,B, 3A,B). No significant effects of year, genotype, and their interaction were observed for *Fusarium*. *Cercospora* was not affected by genotype or year × genotype interactions. Since year × genotype was significant for some seed quality components and not for others, we analyzed the data by year to consider the environmental effects (rainfall and temperature).

**Table 2:** **Analysis of variance results for soybean seed yield, seed composition, seed quality, and seed disease measures for 16 soybean genotypes grown under dryland conditions in a 2-year field experiment at Stoneville, MS in 2012 and 2013**.

**Trait**	**Year**	**Genotype**	**Year × genotype**	**Residuals**
DF	1	15	15	
Seed yield (kg/ha)	11.1^*^	4.6^***^	1.84^*^	2,60,877
100 seed weight (g per100)	19.7^*^	19.3^***^	ns	0.57
Protein	63.9^***^	67.7	2.81^***^	0.49
Oil	12.4^*^	39.0^***^	3.5^***^	0.21
**FATTY ACIDS**				
Palmitic	173.8^***^	10.1^***^	3.73^***^	0.07
Stearic	56.2^**^	6.0^***^	1.9^*^	0.01
Oleic	343^***^	38.1^***^	5.3^***^	0.88
Linoleic	251^***^	30.2^***^	ns	0.65
Linolenic	31.8^**^	16.7^***^	4.7^***^	0.17
**MACRO-NUTRIENTS**				
Ca	25.9^**^	65.3^***^	5.46^***^	0.0006
K	13.0^*^	25.4^***^	5.9^***^	0.009
Mg	15.3^*^	14.2^***^	3.4^***^	0.0001
P	105^***^	24.5^***^	ns	0.0006
C	995^***^	13.9^***^	2.8^**^	0.11
N	17.5^*^	31.5^***^	4.1^***^	0.025
S	295^***^	6.7^***^	ns	0.0002
**MICRO-NUTRIENTS**				
B	16.1^*^	70.1^***^	2.9^**^	2.62
Cu	71.6^***^	47.2^***^	2.7^**^	0.49
Fe	125^***^	4.3^***^	ns	23.46
Mn	21.3^**^	9.2^***^	ns	2.53
Mo	10.2^*^	47.5^***^	ns	0.52
Zn	127^***^	10.9^***^	2.5^**^	7.52
**SEED QUALITY**				
Germination	7.4^*^	21.6^***^	ns	87.42
Accelerated aging	23.0^**^	15.5^***^	3.6^***^	96.59
Total seed damage	ns	4.5^***^	6.5^***^	0.098
Heat damage	ns	ns	ns	0.003
Hard seed	ns	17.2^***^	2.7^**^	17.26
Wrinkling	13.5^*^	14.7^***^	2.6^**^	33.19
Green seed	ns	15.0^***^	4.47^***^	58.33
**SEED DISEASE**				
Mold	ns	ns	ns	32.29
Purple seed	4.0^*^	4.0^***^	4.0^***^	1.04
Phomopsis	61.0^***^	2.5^**^	1.99^*^	33.67
Cercospora	10.0^**^	ns	ns	7.33
Fusarium	ns	ns	ns	61.98
Charcoal rot	ns	1.8^*^	1.8^*^	0.49

* Significance at P ≤ 0.05;

** significance at P ≤ 0.01;

*** *significance at P ≤ 0.001*.

### Seed germinability, yield, and composition (protein, oil, and fatty acids)

Yield showed significant differences between years. Yield in 2012 was higher than in 2013, except for AG5606 (Table 3), which had an 8.6% increase in 2013. For the other genotypes, the difference in yield between years ranged from a 12.7 to a 58.8% decrease in 2013 over 2012 (Table 3). Two breeding lines (one MS and one IL breeding line) were more stable for yield, with each line having < 15% difference in yield between years (Table 3). The MS breeding line, 25-1-1-4-1-1, yielded higher than all other breeding lines and higher than all cultivars except AG3803, AG3905, and DK4866 in 2013, the more stressful year. Although 2013 was generally cooler than 2012 (Figures 2A,B, 3A,B), rainfall was more uniform during the growing season in 2012 than in 2013 and also more rainfall occurred in July through August (seed-fill stage) in 2012 (Figures 1A,B). It appears that the higher yield in 2012 was likely due to rainfall uniformity and higher rainfall during the seed-fill stage. However, this cannot be generalized, as the higher yield of AG5606 (8.6% higher) in 2013 may be due to its longer maturity (Table 1), which may have better utilized the August and September rains occurring in that year.

**Table 3:** **Percentage differences in yield (kg/ha), germination (%), and phomopsis (%) between 2012 and 2013 in each genotype**.

**Genotype**	**Yield (kg/ha)**			**Germination (%)**			**Phomopsis (%)**		
	**2012**	**2013**	**Diff^c^**	**2012**	**2013**	**Diff**	**2012**	**2013**	**Diff**
**MS BREEDING LINES**^a^									
04025-41	2578	1807	771	57.3	81.7	−24.3	20	0	20.0
24-2-1-2-1-2	2679	1585	1094	70.7	91.3	−20.7	28	1.3	26.7
25-1-1-4-1-1	2741	2372	370	87.3	94.3	−7.0	9.3	0	9.3
34-3-1-2-4-1	2602	1991	612	92.0	96.0	−4.0	5.3	0	5.3
**CULTIVARS**									
AG3803	3687	2865	822	36.3	50.3	−14.0	2.7	0	2.7
AG3905	3797	2670	1128	55.0	78.7	−23.7	12	4	8.0
AG4403	3692	2345	1348	70.7	78.0	−7.3	13.3	1.3	12.0
AG4903	3690	2150	1540	43.0	51.7	−8.7	5.3	0	5.3
AG5606	2114	2295	−182	47.3	47.3	0.0	8	0	8.0
C4926	2509	1722	787	41.7	54.7	−13.0	6.7	0	6.7
DK4866	4122	2428	1695	34.0	41.0	−7.0	14.7	5.3	9.4
Dwight	2374	1766	608	49.0	58.7	−9.7	16	0	16.0
LD00-3309	3405	1403	2002	51.0	44.3	6.7	1.3	0	1.3
**IL BREEDING LINES**^b^									
LG03-4561-14	2571	2246	325	60.3	83.0	−22.7	10.7	2.7	8.0
LG03-4561-19	2990	2096	895	65.0	70.0	−5.0	8	1.3	6.7
LG04-1459-6	3775	2314	1462	80.0	81.7	−1.7	5.3	2.7	2.6
LSD	256	403		5.1	6.1		4.4	1.2	
Min	2114	1403	−182	34.0	41.0	−24.3	1.3	0.0	1.3
Max	4122	2865	2002	92.0	96.0	6.7	28.0	5.3	26.7
Average	3083	2128	955	58.8	68.9	−10.1	10.4	1.2	9.3
Median	2866	2198	858	56.2	74.0	−8.0	8.7	0.0	8.0

a Percentage exotic of 50%;

b Percentage exotic ranged from 19 to 25%;

c *Diff, Difference between year 2012 and 2013. The experiment was conducted in 2012 and 2013 at Jamie Whitten Delta States Research Center, Stoneville, MS*.

Three genotypes (25-1-1-4-1-1, 34-3-1-2-4-1, and LG04-1459-6, had germination rates of ≥80% in both years (Table 3). The protein content of the first two genotypes was significantly higher in both years (>40%) than that of all nine cultivars (Table 4). However, LG04-1459-6 had the lowest protein level (36.1% in 2012 and 34.7% in 2013; Table 4) of any genotype tested. This apparent inconsistency between protein and germination is further indicated by the non-significant correlations between germination and protein and oil levels (Table 5). This may indicate that protein level *per se* may not be important for high germination. It may also coincidentally reflect a naturally occurring high protein level in PI 587982A, the parent of both of the above two lines with both high germination and high protein. The association of high germinability and high protein likely needs further study. Among the highest oleic levels in the trial was in AG4403 (32.7% in 2012 and 27.7% in 2013), which had moderate germination percentages of 70.7% in 2012 and 78.0% in 2013. This may indicate that oleic acid level does not have a strong association with germination. Generally, the accumulation of protein and oleic acid was higher in 2012 than in 2013. However, the accumulation of palmitic and linoleic acid was higher only in 2013 (Table 4).

**Table 4:** **Soybean seed composition constituents (protein, oil, and fatty acids, %) of breeding lines and cultivars under dryland conditions**.

**Genotype**	**Protein (%)**		**Oil (%)**		**Palmitic (%)**		**Stearic (%)**		**Oleic (%)**		**Linoleic (%)**		**Linolenic (%)**	
	**2012**	**2013**	**2012**	**2013**	**2012**	**2013**	**2012**	**2013**	**2012**	**2013**	**2012**	**2013**	**2012**	**2013**
**MS BREEDING LINES**^a^														
04025-41	42.5	40.3	21.0	21.6	10.1	11.4	4.2	4.5	33.8	28.1	50.7	53.1	4.3	4.3
24-2-1-2-1-2	42.3	41.2	20.7	21.4	10.6	12.0	4.0	4.4	30.3	23.3	51.4	55.2	4.8	6.6
25-1-1-4-1-1	43.3	40.1	19.3	20.0	10.3	11.6	3.9	4.3	31.8	26.0	51.4	54.4	4.8	4.4
34-3-1-2-4-1	43.6	41.2	20.5	20.1	10.3	11.9	4.3	4.6	32.4	31.4	48.8	51.9	5.6	5.2
**CULTIVARS**														
AG3803	38.4	38.4	22.6	21.8	10.2	10.8	4.3	4.4	28.2	25.0	53.0	55.9	5.4	6.3
AG3905	38.5	38.1	21.8	20.9	10.3	10.8	4.2	4.4	27.9	26.6	52.5	55.5	5.2	5.4
AG4403	36.9	36.0	23.8	22.8	10.5	11.1	4.4	4.5	32.7	27.7	51.5	53.6	3.6	5.2
AG4903	38.9	37.2	21.3	21.1	9.7	11.0	4.2	4.5	27.8	22.7	54.4	57.4	4.7	6.5
AG5606	39.8	37.7	20.3	20.4	10.1	11.3	3.9	4.5	26.1	21.5	55.1	57.5	6.0	7.3
C4926	37.2	36.5	21.5	21.1	10.3	11.0	4.3	4.6	28.3	23.5	53.9	57.1	4.9	6.3
DK4866	37.3	36.4	21.2	20.9	9.5	10.5	4.1	4.4	27.4	22.7	55.8	57.9	5.4	6.8
Dwight	38.3	37.5	22.3	21.8	10.6	11.1	4.4	4.6	27.0	23.8	53.7	56.2	5.2	6.4
LD00-3309	36.8	37.1	22.6	21.1	10.4	11.2	4.3	4.7	25.8	23.9	54.9	56.5	5.4	5.8
**IL BREEDING LINES**^b^														
LG03-4561-14	35.7	35.4	23.8	22.9	10.1	11.0	4.2	4.5	27.3	23.6	54.2	56.4	5.7	7.1
LG03-4561-19	35.8	35.2	23.8	22.5	10.3	10.6	4.2	4.4	26.7	25.0	53.8	56.0	6.0	6.6
LG04-1459-6	36.1	34.7	23.8	22.8	9.8	10.5	4.1	4.4	27.2	23.8	55.0	57.5	5.6	6.2
LSD	0.33	0.45	0.16	0.33	0.14	0.15	0.06	0.07	0.62	0.42	0.55	0.32	0.28	0.21

a MS breeding lines, Mississippi breeding lines;

b *IL breeding lines, Illinois breeding lines*.

*The experiment was conducted in 2012 and 2013 at Jamie Whitten Delta States Research Center, Stoneville, MS*.

**Table 5:** **Correlation between germination and seed quality components in 16 soybean genotypes grown under dryland conditions in a 2-year field experiment**.

**Trait**	**2012 ***R***-Value**	**Significance**	**2013 ***R***-Value**	**Significance**
Yield	−0.21	ns	−0.01	ns
Protein	0.43	ns	0.42	ns
Oil	−0.07	ns	0.08	ns
Oleic	0.53	^*^	0.62	^*^
Palmitic	0.33	ns	0.52	^*^
Linolenic	−0.07	ns	−0.52	^*^
Linoleic	−0.63	^**^	−0.70	^**^
P	0.49	ns	0.58	^*^
B	0.37	ns	0.54	^*^
Cu	0.51	^*^	0.59	^*^
Mo	0.46	ns	0.62	^**^
N	0.42	ns	0.55	^*^
K	0.53	^*^	0.54	^*^
Ca	−0.76	^***^	−0.61	^*^
Hard seed	−0.81	^***^	−0.95	^***^
Wrinkle	−0.69	^**^	−0.47	ns
Green seed	−0.34	ns	−0.60	^*^
Accelerated aging (AA)	0.54	^*^	0.73	^**^

* Significance at P ≤ 0.05;

** significance at P ≤ 0.01;

*** *significance at P ≤ 0.001*.

*The genotypes differed in their germinability. The experiment was conducted in 2012 and 2013 at the Jamie Whitten Delta States Research Center, Stoneville, MS*.

### Seed germinability and seed macro- and micro-nutrients

Germination ranged from a 13.1% decrease in 2013 over 2012 for LD00-3309 to a 43% increase for AG3905 (Table 3). Except for LD00-3309 and AG5606, germination was higher in 2013 than in 2012. For example, in 2012 only three genotypes (25-1-1-4-1-1, 34-3-1-2-4-1, and LG04-1459-6) had germinations ≥80%, while in 2013 the number doubled and included 25-1-1-4-1-1, 34-3-1-2-4-1, LG04-1459-6, 04025-41, 24-2-1-2-1-2, and LG03-4561-14. The former three were ≥80% in 2012 and in 2013, while the latter three went from germinations of 57, 60, and 71% in 2012 to 82, 83, and 91% in 2013, respectively. The higher germinations for most genotypes in 2013 could be due to the cooler temperature during the seed-fill period, especially between July and early August in 2013 (Figures 2A,B, 3A,B). Temperature data showed that the maximum and minimum air temperatures in 2013 were lower than in 2012 during May through July. For example, in June the maximum temperatures were 31.7 vs. 30.4°C, respectively in 2012 and 2013. In July the maximum temperatures were 33.7 vs. 31.4°C, respectively in 2012 and 2013. The same pattern was shown for the minimum temperatures (Figures 2A,B). It was interesting that although germination was generally higher in 2013 for most genotypes, yield was generally higher in 2012. For most lines, the environment that produced the highest yield was not the one that produced the highest seed germination.

Seed content of macro-nutrients P and N were significantly higher in two of the genotypes with ≥80% germination in both years (25-1-1-4-1-1and 34-3-1-2-4-1) than in the other genotypes, except for 04025-41 and 24-2-1-2-1-2, which performed similar to the above two high germinability genotypes (Table 6). Seed K was significantly higher in 25-1-1-4-1-1 and 34-3-1-2-4-1 than in the rest of the genotypes, except 04025-41, 24-2-1-2-1-2, LG04-1459-6, and AG5606. Calcium content was significantly lower in two of the genotypes with ≥80% germination in both years (25-1-1-4-1-1 and 34-3-1-2-4-1). The same was true for 04025-41 and 24-2-1-2-1-2, which performed similar to the above two high germinability genotypes for macro- and micro-nutrient content. There was no consistent trend for Mg, C, and S. Accumulation of Ca, P, C, N, and S in 2012 was almost always higher than in 2013 (Table 6). Seed content of micro-nutrients B, Cu, and Mo was significantly higher in two of the genotypes with ≥80% germination (25-1-1-4-1-1 and 34-3-1-2-4-1) in both years than in the other genotypes. Again, genotypes 04025-41 (germination rate of 57.3% in 2012 and 81.7% in 2013) and 24-2-1-2-1-2 (germination rate of 70.7% in 2012 and 91.3% in 2013), tended to perform similarly to the above two high germinability genotypes with respect to B, Cu, and Mo. Nutrients Fe, Mn, and Zn had no clear consistent trend between genotypes. The accumulation of B, Cu, Fe, Mo, and Zn was almost always higher in 2013 than in 2012 (Table 7). There were significant correlations (*P* ≤ 0.05) between germination and Ca, Cu, and K in both years and for P, B, Mo, and N only in 2013 (Table 5). The lower nutrients (protein and macro-nutrients) in LG04-1459-6 in spite of its ≥80% germination in both years, could be due to the difference of parental lines between LG04-1459-6 in one hand, and 25-1-1-4-1-1 and 34-3-1-2-4-1 on the other hand.

**Table 6:** **Soybean seed macro-nutrients (%) in breeding lines and cultivars under dryland conditions**.

**Genotype**	**Ca (%)**		**K (%)**		**Mg (%)**		**P (%)**		**C (%)**		**N (%)**		**S (%)**	
	**2012**	**2013**	**2012**	**2013**	**2012**	**2013**	**2012**	**2013**	**2012**	**2013**	**2012**	**2013**	**2012**	**2013**
**MS BREEDING LINES**														
04025-41	0.26	0.25	2.32	2.27	0.20	0.24	0.60	0.65	49.0	53.1	6.35	6.52	0.26	0.33
24-2-1-2-1-2	0.21	0.36	2.24	2.07	0.23	0.26	0.57	0.64	49.0	52.9	6.39	6.40	0.28	0.32
25-1-1-4-1-1	0.23	0.27	2.25	2.08	0.21	0.24	0.63	0.63	48.4	52.5	6.21	6.56	0.26	0.33
34-3-1-2-4-1	0.28	0.36	2.37	2.15	0.23	0.25	0.59	0.64	49.1	52.4	6.37	6.57	0.29	0.33
**CULTIVARS**														
AG3803	0.42	0.40	1.70	1.95	0.24	0.24	0.48	0.52	50.0	53.6	5.64	5.27	0.27	0.33
AG3905	0.40	0.47	1.57	1.86	0.25	0.27	0.50	0.56	49.6	52.9	5.70	5.50	0.26	0.32
AG4403	0.38	0.44	1.47	1.85	0.23	0.26	0.46	0.55	50.3	53.3	5.63	5.71	0.24	0.31
AG4903	0.43	0.43	1.88	1.91	0.25	0.25	0.49	0.56	49.2	52.8	5.75	6.00	0.26	0.33
AG5606	0.39	0.36	1.85	2.05	0.23	0.25	0.51	0.57	49.0	52.8	5.87	5.65	0.26	0.31
C4926	0.41	0.42	1.73	1.81	0.21	0.23	0.50	0.56	49.1	53.1	5.51	5.76	0.25	0.31
DK4866	0.49	0.50	1.70	1.92	0.28	0.27	0.47	0.53	49.2	52.7	5.52	5.75	0.23	0.29
Dwight	0.42	0.46	1.71	1.88	0.28	0.27	0.51	0.53	49.9	53.1	5.65	5.98	0.25	0.30
LD00-3309	0.50	0.56	1.66	1.93	0.29	0.27	0.49	0.55	49.5	52.3	5.49	6.06	0.24	0.30
**IL BREEDING LINES**														
LG03-4561-14	0.38	0.42	1.93	2.05	0.26	0.26	0.52	0.53	50.4	53.5	5.35	5.76	0.26	0.31
LG03-4561-19	0.34	0.40	1.83	2.04	0.26	0.25	0.51	0.55	50.6	53.4	5.34	5.68	0.25	0.30
LG04-1459-6	0.32	0.37	1.75	2.05	0.25	0.25	0.44	0.51	50.6	53.6	5.33	5.80	0.23	0.29
LSD	0.02	0.01	0.06	0.05	0.01	0.01	0.01	0.01	0.20	0.19	0.08	0.10	0.01	0.01

*MS breeding lines, Mississippi breeding lines; IL breeding lines, Illinois breeding lines*.

*The experiment was conducted in 2012 and 2013 at the Jamie Whitten Delta States Research Center, Stoneville, MS*.

**Table 7:** **Soybean seed micro-nutrient concentrations (mg/kg) in breeding lines and cultivars under two dryland environments**.

**Genotype**	**B (mg/kg)**		**Cu (mg/kg)**		**Fe (mg/kg)**		**Mn (mg/kg)**		**Mo (mg/kg)**		**Zn (mg/kg)**	
	**2012**	**2013**	**2012**	**2013**	**2012**	**2013**	**2012**	**2013**	**2012**	**2013**	**2012**	**2013**
**MS BREEDING LINES**^a^												
04025-41	41.2	44.0	19.5	19.9	65.5	87.3	21.2	21.9	7.4	9.9	52.0	60.2
24-2-1-2-1-2	42.5	40.9	19.5	20.5	67.3	83.6	19.6	21.9	6.5	9.1	56.6	63.3
25-1-1-4-1-1	41.9	41.3	20.3	20.1	67.4	81.7	16.6	18.5	9.4	10.6	57.6	61.8
34-3-1-2-4-1	42.8	42.1	18.5	20.1	69.1	88.3	21.9	24.6	7.5	9.5	56.1	61.1
**CULTIVARS**												
AG3803	32.7	37.2	13.4	15.7	60.9	75.1	21.1	25.2	3.7	4.5	42.9	53.1
AG3905	28.3	33.2	14.9	17.9	69.2	85.5	21.5	26.9	3.6	5.0	48.7	61.0
AG4403	27.1	31.9	13.2	16.2	59.1	81.2	19.3	22.5	3.5	4.7	45.3	60.2
AG4903	30.3	31.7	14.7	17.3	67.5	81.7	21.0	23.3	4.5	5.2	50.3	62.9
AG5606	33.0	33.7	17.1	18.3	70.1	88.8	18.5	20.9	6.1	6.1	48.5	56.3
C4926	38.5	39.8	14.5	16.5	65.3	77.1	21.3	22.7	4.7	5.9	51.2	60.9
DK4866	30.0	31.5	14.8	17.2	66.1	82.0	20.7	24.3	4.4	5.1	50.1	61.1
Dwight	26.9	28.1	14.9	16.0	76.1	88.8	24.0	23.9	3.0	4.4	50.2	63.9
LD00-3309	25.9	30.4	15.3	16.9	70.1	82.5	23.4	25.9	3.1	4.7	48.3	65.3
**IL BREEDING LINES**^b^												
LG03-4561-14	33.2	36.6	15.1	16.3	67.6	81.4	20.6	21.1	4.1	5.0	47.5	57.0
LG03-4561-19	30.4	36.1	14.5	15.6	67.3	89.0	19.9	20.6	3.5	4.8	45.9	59.1
LG04-1459-6	25.9	29.3	14.6	17.4	58.9	75.9	17.4	20.7	3.1	4.5	41.3	53.4
LSD	1.04	0.86	0.35	0.44	2.80	2.79	1.03	0.80	0.44	0.52	1.47	1.74

a MS breeding lines, Mississippi breeding lines;

b *IL breeding lines, Illinois breeding lines*.

*The experiment was conducted in 2012 and 2013 at Jamie Whitten Delta States Research Center, Stoneville, MS*.

### Seed germinability, plant and seed physical characteristics, and fungal infection

**Figure 4:**
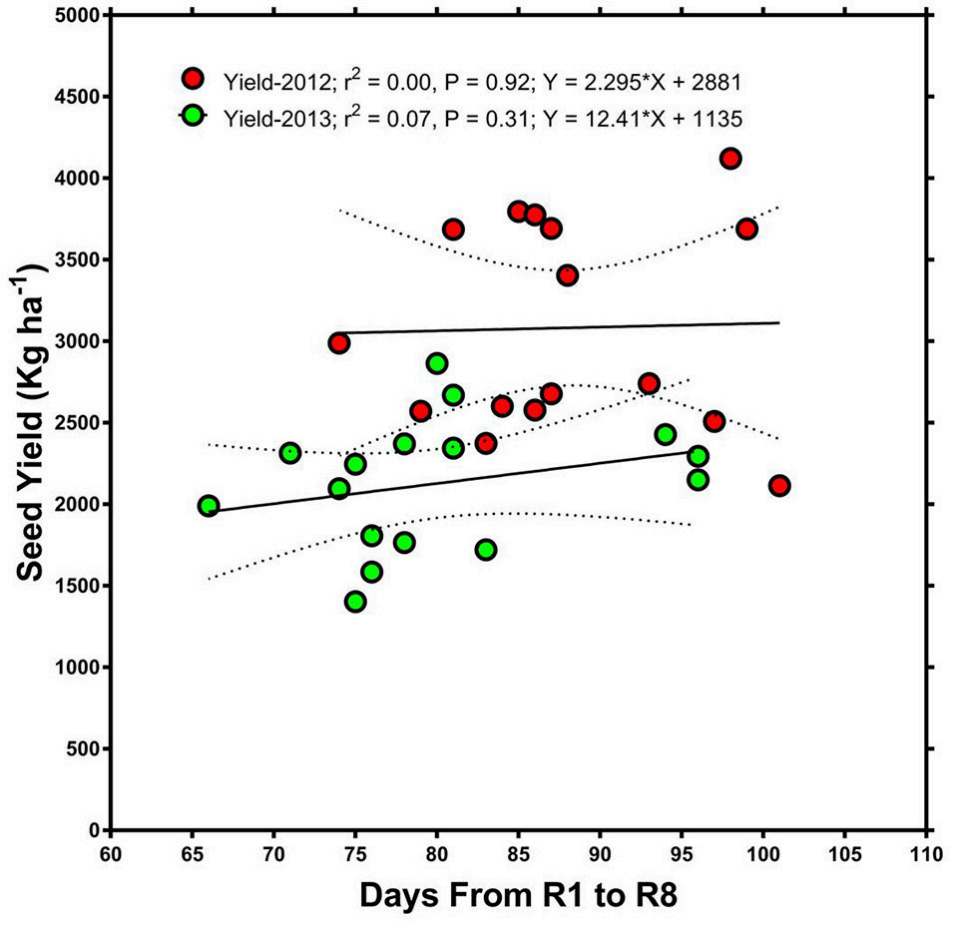
**Relationship between days after planting (time from beginning of flowering, R1, to full maturity stage, R8) and seed yield**. The graph shows that there was no significant relationship between the time from R1 to R8 and seed yield in either 2012 or 2013.

Beginning bloom (R1), harvest date, and full maturity (R8) were reached later in 2013 than in 2012 (Table 1). This was probably mostly due to the later planting date in 2013 (30 April compared to 13 April in 2012), but may also have been influenced by rainfall and temperature differences between years. For example, rainfall and temperature amounts and patterns were different in each year (Figures 1–3). Even though R8 was reached later in 2013, over all genotypes, the time from R1 to R8 was longer in 2012 than in 2013 (ranging from 1 to 18 days longer with an average of 8 days longer). However, there was no significant (*P* > 0.05) correlation between the time from R1 to R8 and seed yield in either 2012 or 2013. Also, there was no significant (*P* > 0.05) correlation between the time from R1 to R8 and seed germination in 2012 but there was in 2013 (*R* = 0.45; *P* = 0.0043). Cause and effect relationship (regression) between the time from R1 to R8 and seed yield showed no significant effects (Figure 4).

### Correlation between germination and seed quality components

There was a positive correlation between germination and oleic acid and a negative correlation between germination and linoleic acid in both years (Table 5). There was a negative correlation between germination and linolenic acid and a positive correlation between germination and palmitic acid in 2013 only. A positive correlation between germination and K and Cu was observed in both years, but positive correlations between germination and B and germination and Mo were shown in 2013 only. A negative correlation was shown between germination and Ca and germination and hard seed in both years (Table 5).

## Discussion

### Seed germinability, yield, and composition

The mechanisms of how seed protein or oleic acid affects germination are still lacking. For example, LeVan et al. (2008) studied the effect of seed composition and seed moisture on germination under a controlled environment and under field conditions and concluded that seed composition may play an important role in imbibitional injury at low seed moisture content. They reported that there was a quadratic relationship between seed protein content and seed germination at different levels of seed moisture, but this relationship was inconclusive for seed oil content and seed germination. They suggested that further research was needed to evaluate the relationship between fatty acids and seed germination. Recently, Chebrolu et al. (2016) studied the effects of three temperature regimes (28/22°C, 36/24°C, and 42/26°C day/night) on heat tolerant line 04025-1-1-4-1-1 (same as 25-1-1-4-1-1 in the current study) and heat-sensitive line DT97-4290 under growth chamber conditions. The germination of seeds of the sensitive genotype had a 50% reduction at 36°C and was completely inhibited at 42°C. By comparison, the tolerant genotype was unaffected at 36/24°C, but had only 25% germination at 42°C. They also found that the heat sensitive genotype accumulated more seed oil at high temperature (42°C), but it did not differ at 28 or 36°C. Protein content of the heat tolerant genotype, as indicated by nitrogen content, did not differ among temperatures (28, 36, and 42°C). Compared with the heat sensitive genotype, the tolerant genotype accumulated more protein at 36 and 42°C, but no difference was observed between genotypes at 28°C. This may indicate reduction in protein synthesis or protein degradation in the heat sensitive genotype under high heat, compared to the heat tolerant genotype, which maintained similar protein levels at all temperatures. Similarly, 25-1-1-4-1-1 had nearly identical protein levels with AG4903 at four locations in the 2011 Uniform Soybean Test—Southern States, Preliminary Maturity Group IV-S-Late (Gillen and Shelton, 2011), but under the likely more heat stressful conditions of the ESPS, 25-1-1-4-1-1 had significantly higher protein content (43.3 and 40.1% for 2012 and 2013, respectively) than AG4903 (38.9 and 37.2%, respectively) in the current study (Table 4).

In the study of Chebrolu et al. (2016), seed oil was higher in the heat sensitive genotype than in the heat tolerant genotype at 42°C, but it was not different between genotypes at 28 or 36°C. They concluded that the higher levels of metabolites such as tocopherols, flavonoids, phenylpropanoids, and ascorbate precursors in the heat tolerant genotype at both temperatures could partially be responsible for the observed heat tolerance in 25-1-1-4-1-1. Our results are partially in agreement, as oil was not correlated with germination, and the daily high temperature in the field was about 36°C, but did not reach 42°C as in the study of Chebrolu et al. (2016). Further research is needed to evaluate the relationship between fatty acids and seed germination, as the relation between oil and germination is still inconsistent (LeVan et al., 2008). The change in seed composition constituents in 2012 and 2013 is in agreement with previous research indicating that temperature was considered a contributing factor to the variability of seed composition, and the increase or decrease of seed oil or protein concentration was associated with the range of temperatures under which soybean seeds mature (Bellaloui et al., 2009a). In our study, 2013 was drier than 2012, especially in July (coinciding with seed-fill for the early maturing genotypes (Figures 1A,B). Furthermore, 2012 was warmer than 2013 (Figures 2A,B). Therefore, the patterns of both rainfall and temperature were different in each year (Figures 2A,B, 3A,B). Hence, it is likely that most of the changes in seed constituents between years were due to drought (Lamoine et al., 2013; Andrade et al., 2016) and heat (LeVan et al., 2008; Chebrolu et al., 2016).

### Seed germinability and seed macro- and micro-nutrients

Previous research showed that PI 587982A and PI 603723 (genotypes with high seed germination under high temperatures) had higher germination rates and lower hard seed compared to the poor seed germination genotypes PI 80480, PI 84976-1, DSR-3100 RR STS, and Pella 86 (Bellaloui et al., 2012b). Orazaly et al. (2014) observed that Ca content in the seed coat had an effect on water absorption. Other studies found Ca to be negatively correlated with water absorption (Saio et al., 1973; Saio, 1976) and positively correlated with hard seed (Zhang et al., 2009), and that low water absorbance resulted in hard seed and low germination rate. Low Ca and hard seed are important because they affect the texture of soybean natto (Mullin and Xu, 2001), are positively correlated with cooked seed hardness, and are influenced by environmental factors such as temperature and soil type (Chen et al., 2001). The role of Ca in hard seed resides in the important role of Ca in the cell membrane and its contribution to cell wall thickness due to a calcium oxalate cell layer in the cell wall (Webb, 1999). In the current study, Ca was found to be significantly (*P* < 0.05) negatively correlated with germination in both years (*R* = −0.76 and −0.61 in 2012 and 2013, respectively; Table 5). However, Ca was significantly (*P* < 0.01) positively correlated with hardseededness (*R* = 0.81 and 0.68 in 2012 and 2013, respectively).

The significantly higher K, P, N, B, Cu, and Mo content in seeds of 25-1-1-4-1-1 and 34-3-1-2-4-1, compared to all other genotypes with = 80% germination in both years, indicates that these nutrients might be important for germination. If this is true, maintaining higher levels of these nutrients in soil and seed could contribute to germination and overall seed health. Bishnoi et al. (2007) studied the effects of Ca and P on soybean seed production and quality and found that the application of Ca at 100 and P at 90 kg/ha significantly improved soybean seed yield and quality (germination and vigor), but seed quality decreased due to weathering and diseases if harvest was delayed (Bellaloui et al., 2012a). Bishnoi et al. (2007) also found that seed quality (viability and germination %) was enhanced with application of Ca at 100 and P at 90 kg/ha in comparison to plots without application. The positive response of soybean to Ca application in the study of Bishnoi et al. (2007) could be due to Ca deficiency in the soil or reduced Ca supply, as reduced Ca supply to the plant may reduce seed Ca concentration, resulting in poorer seed germination (Keiser and Mullen, 1993). The genotypes used in the current study were different from those used by Bishnoi et al. (2007), which may partially explain the apparent differences in the effect of Ca between the current study and that of Bishnoi et al. (2007).

The significant role of macro-nutrients such as N, P, K, S, and Ca and micro-nutrients such as Fe, Zn, and B to plant growth, development, yield, and quality has been well-documented (Mengel and Kirkby, 1982; Marschner, 1995; Samarah and Mullen, 2004). For example, Haq and Mallarino (2005) reported that N, P, K, and other nutrients can affect several physiological processes that, in turn, could affect grain yield and protein or oil content. Different levels of seed nutrients among genotypes could be due to genotypic background differences and to their different responses to environment, especially drought and heat. It was previously reported that the content of seed micro- and macro-nutrients was found to be influenced by environment and genotype (Zhang et al., 1996; Haq and Mallarino, 2005; Bellaloui et al., 2011, 2015).

### Seed germinability, plant and seed physical characteristics, and fungal infection

Seed physical characteristics (quality) and seed diseases are shown in Tables 8, 9, respectively. The response of genotypes for hard seed differed in each year, indicating the contribution of both genotypic and environmental factors to this trait. In terms of environment, 2013 produced a higher level of hardseededness than 2012 for most genotypes. In terms of genotypic effect, the four breeding lines derived from PIs 587982A and 603756 (the MS breeding lines) averaged < 1% hard seed in both 2012 and 2013, whereas the level of hard seed of the nine cultivars ranged from 1.3 to 17.0% in 2012 and from 4.0 to 27.7% in 2013 (Table 8). Clearly, there was a major genotypic effect. Recently, a major single recessive gene (*isc*) for permeable seed coat was identified in PI 587982A (Kebede et al., 2014), whose permeable seed coat effect can be observed in the PI 587982A-derived lines 04025-41, 25-1-1-4-1-1, and 34-3-1-2-4-1 (Table 8).

**Table 8:** **Soybean seed quality components (%) in breeding lines and cultivars under two dryland environments**.

**Genotype**	**Wrinkling**		**Accelerated aging**		**Total seed damage**		**Hard seed**		**Green seed**	
	**2012**	**2013**	**2012**	**2013**	**2012**	**2013**	**2012**	**2013**	**2012**	**2013**
**MS BREEDING LINES**^a^										
04025-41	30.0	13.3	26.3	54.3	0.53	0.40	0.0	0.7	0.0	0.0
24-2-1-2-1-2	20.0	10.0	71.7	69.3	0.33	0.27	0.3	0.0	0.0	10.0
25-1-1-4-1-1	13.3	3.3	82.7	90.3	0.77	0.23	0.7	0.3	0.0	0.0
34-3-1-2-4-1	10.0	0.0	87.3	94.7	0.30	0.23	0.0	0.0	0.0	0.0
**CULTIVARS**										
AG3803	30.0	26.7	19.3	55.7	0.27	0.40	12.0	19.7	50.0	26.7
AG3905	23.3	26.7	38.3	66.3	0.47	0.20	2.7	4.7	20.0	6.7
AG4403	20.0	23.3	39.0	69.7	0.13	0.27	1.3	4.0	23.3	10.0
AG4903	30.0	23.3	38.3	54.0	0.50	0.33	13.0	16.3	10.0	16.7
AG5606	30.0	20.0	30.3	32.3	0.43	0.23	10.3	12.3	3.3	26.7
C4926	50.0	40.0	51.0	68.3	0.70	0.27	14.0	7.3	16.7	33.3
DK4866	30.0	20.0	64.3	55.3	0.60	1.37	5.3	22.0	0.0	20.0
Dwight	26.7	20.0	49.3	69.7	1.30	0.10	15.3	10.7	20.0	6.7
LD00-3309	16.7	10.0	59.3	62.0	0.47	0.13	17.0	27.7	26.7	23.3
**IL BREEDING LINES**^b^										
LG03-4561-14	20.0	26.7	37.0	79.0	0.30	0.13	0.3	2.3	40.0	30.0
LG03-4561-19	16.7	26.7	58.7	70.0	0.53	0.20	6.0	8.3	26.7	33.3
LG04-1459-6	26.7	16.7	39.3	75.7	0.23	2.10	0.7	5.0	10.0	13.3
LSD	3.1	3.4	5.8	5.9	0.18	0.20	1.7	3.0	3.6	4.8

a MS breeding lines, Mississippi breeding lines;

b *IL breeding lines, Illinois breeding lines*.

*The experiment was conducted in 2012 and 2013 at Jamie Whitten Delta States Research Center, Stoneville, MS*.

**Table 9:** **Soybean seed diseases (infection %) in breeding lines and cultivars under two dryland environments**.

**Genotype**	**Mold (%)**		**Purple seed (%)**		**Phomopsis (%)**		**Cercospora (%)**		**Fusarium (%)**		**Charcoal rot (%)**	
	**2012**	**2013**	**2012**	**2013**	**2012**	**2013**	**2012**	**2013**	**2012**	**2013**	**2012**	**2013**
**MS BREEDING LINE**^a^												
04025-41	0.0	3.3	0.0	0.0	20.0	0.0	4.0	0.0	4.0	0.0	0.0	0.0
24-2-1-2-1-2	0.0	0.0	0.0	0.0	28.0	1.3	1.3	0.0	5.3	0.0	0.0	0.0
25-1-1-4-1-1	0.0	0.0	0.0	0.0	9.3	0.0	4.0	1.3	25.3	2.7	0.0	0.0
34-3-1-2-4-1	0.0	0.0	0.0	0.0	5.3	0.0	0.0	0.0	1.3	0.0	0.0	0.0
**CULTIVARS**												
AG3803	0.0	0.0	0.0	0.0	2.7	0.0	0.0	1.3	1.3	0.0	0.0	0.0
AG3905	0.0	0.0	0.0	0.0	12.0	4.0	0.0	0.0	0.0	0.0	0.0	0.0
AG4403	10.0	0.0	0.0	0.0	13.3	1.3	5.3	2.7	0.0	0.0	0.0	0.0
AG4903	0.0	3.3	0.0	0.0	5.3	0.0	4.0	1.3	1.3	0.0	0.0	0.0
AG5606	0.0	3.3	0.0	0.0	8.0	0.0	4.0	0.0	9.3	1.3	0.0	0.0
C4926	0.0	3.3	0.0	0.0	6.7	0.0	6.7	0.0	0.0	2.7	0.0	0.0
DK4866	0.0	3.3	6.7	0.0	14.7	5.3	8.0	0.0	2.7	1.3	0.0	0.0
Dwight	0.0	13.3	0.0	0.0	16.0	0.0	2.7	1.3	1.3	0.0	1.3	0.0
LD00-3309	0.0	0.0	0.0	0.0	1.3	0.0	0.0	0.0	0.0	1.3	0.0	0.0
**IL BREEDING LINES**^b^												
LG03-4561-14	0.0	0.0	0.0	0.0	10.7	2.7	0.0	1.3	0.0	0.0	1.3	0.0
LG03-4561-19	0.0	0.0	0.0	0.0	8.0	1.3	0.0	0.0	0.0	0.0	2.7	0.0
LG04-1459-6	0.0	3.3	0.0	0.0	5.3	2.7	0.0	2.7	0.0	1.3	0.0	0.0
LSD	2.4	3.8	0.8	0.0	4.4	1.2	2.0	0.9	6.1	1.1	0.6	0.0

a MS breeding lines, Mississippi breeding lines;

b *IL breeding lines, Illinois breeding lines*.

*The experiment was conducted in 2012 and 2013 at Jamie Whitten Delta States Research Center, Stoneville, MS*.

Total seed damage is the official total measure of grain damage as prescribed by the United States Federal Grain Inspection Service (FGIS). It includes grain damage due to multiple factors, including mold, heat, green seed, stink bug, etc. Grain elevators assess discounts on the value of grain produced by soybean producers based on FGIS standards. This can result in a loss of revenue to producers when they sell their grain. A common level of grain damage that could result in discounting at grain elevators is the 2% level, meaning that damage >2% would result in discounting of payments to producers. In the current study, one Illinois-derived breeding line (LG04-1459-6) had damage >2% (2.1%) in 1 year (2013; Table 8). All other lines had total grain damage of < 2% in 2012 and 2013 and so would not have been assessed damage charges under the conditions of this study.

Seed coat wrinkling is a type of seed damage that is measured by FGIS standards, but is not discounted by elevators. Even so, it has been shown to be negatively correlated with seed germination and seed vigor (Smith et al., 2008). As with many physical seed characteristics, it is influenced by both environment and genotype. In the current study, most genotypes showed higher wrinkling in 2012 than in 2013 (Table 8), indicating an effect due to environment. Recent studies have also shown a genetic effect involved in the level of seed coat wrinkling. Kebede et al. (2013) identified a major single dominant gene (*Wri*) in PI 567743 that controls the level of seed coat wrinkling observed in high heat environments, such as the ESPS. The two MS breeding lines (25-1-1-4-1-1 and 34-3-1-2-4-1) derived from PI 587982A had significantly lower levels of seed coat wrinkling than all other lines tested in both years. For example, cultivar C4926 had wrinkling scores of 50 and 40% in 2012 and 2013, respectively, whereas 25-1-1-4-1-1 had wrinkling scores of 13.3 and 3.3% in those years, respectively (Table 8). Over all genotypes there was a significant negative correlation between germination and wrinkling in 2012 (*R* = −0.69; *P* = 0.01) but not in 2013 (*P* > 0.05) the year with less wrinkling (Table 5).

Green seed damage is assessed by FGIS standards, but in that system there must be a minimum intensity of green before it is reported as damage. Generally, an intensity of light green is not reported as damaged by FGIS standards. The green seed damage estimates for the current study reported any level of green observed. Light green and dark green shades were recorded equally as green seed damage. Green seed damage in soybean is known to be caused by rapid dry down of maturing seed, where the normal degradation of chlorophyll is inhibited (Adams et al., 1983). Hence, any stress (drought, hard freeze, and high heat) that does not allow for the normal slow dehydration and chlorophyll degradation of the seed will promote green seed damage. Rapid dry down of maturing seed, and its resulting green seed damage, is harmful to soybean germination (Green et al., 1965) because it does not allow for the production of the germination-specific enzymes malate synthase and isocitrate lyase (Adams et al., 1983). Hence, environment has a large effect on the level of green seed damage (Green et al., 1965; Adams et al., 1983). In spite of the large potential effect of environment, there was no year effect in the current study (Table 2). Yet, there was a significant year × genotype interaction (Table 2), indicating that some genotypes responded differently to specific within-year environments. This is understandable, given that the 16 genotypes ranged in maturity from MG II to V and experienced maturation under different environmental conditions. For example, MG III AG3905 had 50% green seed damage in 2012, but 26.7% green seed damage in 2013 (Table 8). The earlier planting date (13 April) affected an earlier harvest in 2012 and may have promoted higher green seed damage, as suggested in the study of Green et al. (1965). However, MG IV C4926 matured during different environments in both years and had only 3.3% green seed damage in 2012, but 26.7% in 2013 (Table 8). Green seed damage had a highly significant genotype component in this study (Table 2), indicating that genotypes do not respond the same to the same stress. This can be observed in comparisons between AG3803 and AG3905. Both had similar maturation (Table 1) environments in each year, but AG3803 had no green seed, while AG3905 had high levels, as noted above (Table 8). Likewise, high-germination breeding line 34-3-1-2-4-1 had no green seed damage in either year (Table 8). High-germination breeding line 25-1-1-4-1-1 had 0 and 10% green seed damage in 2012 and 2013, respectively, compared to 3.3 and 26.7% green seed damage for C4926 in 2012 and 2013, respectively (Table 8). Over all genotypes there was not a significant (*P* > 0.05) negative correlation between germination and green seed damage in 2012 but there was in 2013 (*R* = −0.60; *P* < 0.05), the more stressful year (Table 5). More research is needed on the inheritance of tolerance to green seed damage in soybean.

The infection for *C*. *kukuchii* and *Fusarium* spp. was generally low (Table 9). An important exception was the significantly higher level of *Fusarium* spp. observed on 25-1-1-4-1-1 (25.3%) in 2012 (Table 9). This moderately high level of infection, together with the 9% *P. longicolla* infection (Tables 3, 9) may have negatively affected the percent germination of 25-1-1-4-1-1 (87.3%) in 2012 (Table 3), as the germination of 25-1-1-4-1-1 was 94.3 in 2013, when its level of Fusarium was only 2.7% and its level of *P. longicolla* was zero (Table 9). It may seem suprising that these higher levels of fungal infection appeared to have had an insignificant effect on the FGIS total seed damage of 25-1-1-4-1-1, which was 0.77 in 2012 and 0.23 in 2013 (Table 8). But it is frequently observed that seed which appears to have no visible sign of disease will be found to be infested with *P. longicolla* or *Fusarium* spp when plated onto media. FGIS damage ratings are totally visual.

*Phomopsis longicolla* infection (Tables 3, 9) was higher in 2012 than in 2013 and significant differences were found among the lines for *P. longicolla* infestation in 2012. The higher infection in 2012 is most likely due to higher rainfall in 2012 during seed-fill (from August through mid-September), as well as a more positive rain distribution pattern across the growing season (Figures 1, 2). Though germination and *P. longicolla* levels were not significantly correlated (data not shown) it was observed that the 2012 germination of breeding lines 04025-412 and 24-2-1-2-1-2 (57.3 and 70.7%, respectively) were probably negatively impacted by their high *P. longicolla* levels (20 and 28%, respectively) in 2012 as their germination levels in 2013 were much higher (81.7 and 91.3%, respectively).

In some US states, such as Mississippi, the minimum germination for certification is 80% (Keith and Delouche, 1999). Therefore we further evaluated traits based on germination levels of at least 80% within a given environment (year). Comparison of quality components between genotypes with ≥80% and those with < 80% germination identified differences between groups for germination, AA, and Ca in both 2012 and 2013, whereas flowering date, hardseededness, seed coat wrinkling, palmitic acid, N, P, K, B, Cu, and Mo were only significant in 1 year (all in 2013; Table 10). Those traits significantly different only 2013 were probably a result of weather differences between years. The lower Ca was associated with the high-germination group in both years is likely very meaningful (Tables 5, 10) and should be investigated in greater detail. Also of interest is what variables were not different between germination groups in this comparison. Differences in seed yield, seed size, maturity, and total seed damage were not significant in either year between high and low germination groups. This may indicate that genotypes with high germinability could be either low yielding or high yielding, and could be of multiple maturities. Hence, selection for high yield with high germination, and of multiple maturities, should be possible.

**Table 10:** **Difference between lines with <80% germination and those with < 80% germination for plant and seed characteristics**.

**Trait**	**2012**		**2013**	
	Difference^*^	***P*****-Value**	**Difference**	***P*****-Value**
**AGRONOMY/PHYSIOLOGY MEASURES**				
Seed yield (kg/ha)	53.33	0.9012	121.57	0.5700
100 Seed weight (g per100 seed)	1.15	0.2457	1.01	0.1339
Flowering date	−5.03	0.3929	−10.48	0.0288
R8	−4.47	0.6243	−2.06	0.7476
Harvest date	−4.99	0.6162	−1.16	0.8580
**SEED QUALITY (%)**				
Germination	−34.03	0.0004	−30.53	0.0002
Accelerated aging (AA)	−24.93	0.0443	−16.89	0.0233
HS (Hard seed)	13.37	0.0571	22.68	0.0004
Total seed damage (DKT)	0.07	0.6984	−0.21	0.4623
Green seed	14.87	0.1358	11.44	0.0588
Wrinkle	9.74	0.1080	12.00	0.0149
Stinkbug damage	−0.02	0.8037	0.00	0.9362
Mold	0.77	0.6472	1.56	0.4287
**PROTEIN AND OIL (%)**				
Protein	−2.64	0.1294	−1.81	0.0913
Oil	0.83	0.3813	−0.03	0.9535
**FATTY ACIDS (% OF OIL)**				
Palmitic	0.06	0.7647	−0.49	0.0321
Stearic	0.10	0.3161	0.05	0.2964
Oleic	−2.05	0.2206	−1.77	0.1801
Linoleic	1.69	0.1759	1.59	0.0719
Linolenic	−0.23	0.5940	0.62	0.1870
**MACRO-NUTRIENTS (%)**				
C	0.21	0.6469	−0.02	0.9452
Ca	0.11	0.0381	0.11	0.0042
K	−0.31	0.0826	−0.19	0.0004
Mg	0.02	0.2379	0.01	0.2772
N	−0.26	0.2937	−0.53	0.0039
P	−0.05	0.1671	−0.05	0.0249
S	−0.01	0.5864	−0.01	0.1831
**MICRO-NUTRIENTS (mg/kg)**				
B	−4.56	0.2670	−5.68	0.0215
Cu	−2.32	0.1162	−2.31	0.0034
Fe	1.96	0.4980	0.14	0.9560
Mn	2.28	0.0647	2.17	0.0566
Mo	−2.15	0.0779	−3.06	0.0029
Zn	−2.66	0.3864	0.93	0.6247
**SEED DISEASE (%)**				
Cercospora	1.44	0.4262	−0.22	0.6727
Charcoal rot	0.41	0.4243	−	–
Fusarium	−6.94	0.0890	0.00	1.0000
Phomopsis	4.62	0.3104	0.09	0.9224

* *Mean of genotypes with <80% germination minus the mean of genotypes with ≥ 80% germination for each year. In 2012 there were three genotypes with ≥ 80% germination and in 2013 there were six genotypes ≥ 80% germination (see Table 1)*.

*The experiment was conducted in 2012 and 2013 at the Jamie Whitten Delta States Research Center, Stoneville, MS*.

Previous research on seed germination, accelerated aging, hard seed, wrinkling, and diseases (phomopsis and charcoal rot) showed that the high germinating genotypes had the lowest hard seed and seed wrinkling percentages (Smith et al., 2008; Mengistu et al., 2009, 2010). For example, Smith et al. (2008) evaluated seed quality characteristics for 513 soybean lines (486 accessions, 24 ancestors, and cultivars Stalwart, Croton 3.9, and Stressland) with maturity groups ranging from 000 to MG V under field conditions in the early soybean production system at Stoneville, MS, in 2002 and 2003. They found significant (*P* = 0.01) negative correlations between standard germination and hard seed, wrinkled seed, phomopsis, and seed weight. Similar results were found for seed germination and hard seed by Bellaloui et al. (2012b). Also, it was found that *Phomopsis longicolla* Hobbs caused substandard germination (Mengistu and Heatherly, 2006; Smith et al., 2008; TeKrony et al., 1984), and high temperature with wet and dry conditions increased seed coat wrinkling, reducing seed germination (Franca-Neto et al., 1993).

Mengistu et al. (2009) evaluated soybean genotypes of different maturities for seed quality characteristics (seed germination, seed phomopsis infection, hard seed) under different irrigation regimes (non-irrigated, irrigated pre-flowering, and irrigated after flowering). They found that soybean genotypes with higher germination rates had lower phomopsis seed infection, lower hard seed rate, and the germination rate and seed phomopsis infection depended on irrigation type. Therefore, as already noted above, the effect of seed diseases on germination appears to be dependent on genotypic response to the pathogen, severity and threshold of infection, and environmental factors (drought and temperature) and their interactions. Therefore, further research including a larger number of genotypes with higher levels of infestation may show different results.

The causes of poor seed quality in the ESPS were suggested to be related to temperature, soil moisture, and disease infection during the periods from the beginning of seed-fill to full maturity and pre-harvesting, leading to hard seed and low seed viability and vigor (TeKrony et al., 1980; Roy et al., 1994). The differences in hard seed of soybean genotypes have been attributed to genetic variation (Kilen and Hartwig, 1978; Kebede et al., 2014) and environmental conditions (Smith et al., 2008; Mengistu et al., 2009), and may play an important role in preventing phomopsis infection. Hard seed can be considered an undesirable trait, as it lowers seed germination and negatively impacts processing soybean to soy food, which leads to poor quality and adverse cost factors (Mullin and Xu, 2001). The hard seed trait is related to moisture impermeability and seed coat character. The hard seed trait has also been considered a positive trait. Roy et al. (1994) evaluated seed infection in soybean cultivar Forrest (susceptible to infection and with permeable seed coat) and D67-5677-1 (a breeding line with impermeable seed coat). They found that after injecting *Phomopsis longicolla* conidia into the seed cavities of pods that high levels of seed infection occurred in Forrest, but not in the hard seed genotype D67-5677-1. They reported that genotypes D67-5677-1, D86-4629, D86-4565, and D86-4669 (hard seed lines) all expressed resistance to naturally occurring infection by *Phomopsis* during 8 years of evaluations (Roy et al., 1994). They also indicated that impermeable seeds and level of seed infection with *Phomopsis* were negatively correlated. They concluded that although seed coat impermeability *per se* conferred resistance to phomopsis, other research suggested that impermeability alone did not account for the resistance, as much lower correlations were obtained between seed coat impermeability and phomopsis seed infection when a larger number of hard seed genotypes were used (Roy et al., 1994). Clearly, further research is needed to determine the relative contribution of the hard seed trait to disease resistance and seed quality using a larger number of genotypes with more severe disease infection under the stress environmental factors of high heat and water deficit conditions.

### Correlation between germination and seed quality components

Previous research reported the possible involvement of seed composition constituent levels in germination. For example, LeVan et al. (2008) suggested a quadratic relationship between seed protein content and standard seed germination, and found that the variability in seed protein content did not change the quadratic relationship between seed protein content and seed germination. However, they also found that the relationship between seed oil content and seed germination was not conclusive, which was partially supported by our results in that there was no correlation found between germination and protein or oil. Previous research on minerals showed that high germinability breeding lines correlated with seed soluble and structural B (Bellaloui et al., 2008). In this work, B was positively associated with germination in 2013 (*r* = 0.54), but not in 2012 (Table 5). Calcium content in the seed coat was found to be positively correlated with hard seed (Zhang et al., 2009), and influenced by environmental factors such as temperature and soil type (Chen et al., 2001). Smith et al. (2008) evaluated 513 soybean lines and found a significant (*P* = 0.01) negative correlation between standard germination and hard seed (*R* = −0.40), wrinkled seed (*R* = −0.53), phomopsis seed infection (*R* = −0.56), and seed weight (*R* = −0.21), in agreement with our finding for hard seed and wrinkled seed, but not for phomopsis seed infection and seed weight. The lack of correlation of disease infection and seed weight with germination in our experiment could be due to genotype differences as well as environmental factors, especially heat and drought, which may have reduced the incidence of disease. Soybean lines in the current experiment were grown under dryland conditions, differing from those of Smith et al. (2008), where irrigation was applied to alleviate water stress. The severity of diseases such as *Phomopsis* infection to plants was reported to be influenced by environment, involved altered seed composition constituents (Bradley et al., 2002; Bellaloui et al., 2012b), and was affected by temperature, rain/irrigation, genotype, and crop management (Mengistu et al., 2010; Bellaloui et al., 2012a). Research available on the relationship between germination and seed composition constituents is still limited and further research is needed to evaluate the relationship between fatty acids and seed germination (LeVan et al., 2008).

### Overall important discussion points

This study involved soybean genotypes from three distinct germplasm pools; the nine cultivars typify the current pool available to producers, the three breeding lines from the Illinois exotic pool were derived from PIs 68508, 445837, 361064, 407710, 189930, and 68600 and previously selected for high yield potential, and the four breeding lines from the Mississippi exotic pool were derived from PIs 587982A and 603756 and previously selected for high germinability under heat stress. Given the distinct nature of the three germplasm pools, some inter-pool comparisons are in order. First, there was no significant difference between the three pools for seed yield or total seed damage in either year (Table 11). The high germinability (MS) pool had significantly lower seed size than the other two pools in 2013, but not in 2012 (Table 11). The MS pool had lower hard seed and higher germination and AA than the cultivar pool in both years (Table 11). There was less seed wrinkling in the MS pool than in the cultivar pool only in 2013, but the MS pool had lower green seed damage than the other two pools in both years (Table 11). In terms of seed constituents, the MS pool had higher protein than the other two pools in both years, while having lower Ca than the cultivar pool in both years (Table 11). The MS pool had higher oleic acid than the other two pools only in 2012 (Table 11). Given the increasing world-wide demand for high quality protein meal, and given the increased likelihood of global warming, the differences between pools for protein content are striking (Tables 4, 11). During the breeding process, the four lines in the high germinability MS pool were never selected for protein content. Rather, they were repeatedly selected in a pedigree breeding protocol that tested only seed germination from the F_2_ through F_5_ generations. Yet, as can be observed in Table 4, their protein contents are significantly and substantially higher than any other line in either of the other germplasm pools. That all four lines have high protein content is likely due to more than just chance. The potential association between protein content and high germinability needs further investigation.

**Table 11:** **Inter-pool comparisons in the three germplasm pools [(cultivars, Mississippi (MS) exotic breeding lines, Illinoi (IL) exotic breeding lines)]**.

**Year Group**	**2012 Cultivar**	2012 IL breeding^*^lines	**2012 MS breeding lines**	**2013 Cultivar**	**2013 IL breeding lines**	**2013 MS breeding lines**
Kg/ha	3266a	3112a	2650a	2183a	2218a	1939a
g/100 seed	11.3a	12.4a	10.1a	9.8a	10.1a	8.2b
Flowering date	53a	50a	68b	71a	67a	87b
Full maturity (R8)	144ab	129a	155a	155a	144a	161a
Harvesting date	145ab	131a	158a	158a	146a	163a
Hard seed	20.9a	7.8b	0.8b	26.3a	9.3b	0.8b
Germination	47.6b	68.4a	76.8a	56.1b	78.2a	90.8a
Accelerated aging	43.3b	45.0ab	67.0a	59.3b	74.9a	77.2a
Total seed damage	0.54a	0.36a	0.48a	0.37a	0.81a	0.28a
Wrinkle	28.5a	21.1a	18.3a	23.3a	23.3ab	6.7b
Green Seed	18.9a	25.6a	0.0b	18.9a	25.6a	2.5b
Protein	38.0b	35.9c	42.9a	37.2a	35.1b	40.7c
Oil	21.9b	23.8a	20.4c	21.3b	22.7a	20.8b
Palmitic	10.2a	10.1a	10.3a	11.0b	10.7b	11.7a
Stearic	4.2a	4.2a	4.1a	4.5a	4.4a	4.5a
Oleic	27.9b	27.1b	32.1a	24.2a	24.2a	27.2a
Linoleic	53.9a	54.4a	50.6b	56.4a	56.6a	53.7b
Linolenic	5.1a	5.8a	4.9a	6.2a	6.6a	5.1b
Ca	0.43a	0.34b	0.24c	0.45a	0.40ab	0.31b
K	1.70b	1.84b	2.29a	1.91a	2.05b	2.14b
Mg	0.25a	0.25a	0.22b	0.26a	0.25a	0.25a
P	0.49b	0.49b	0.60a	0.55b	0.53b	0.64a
C	49.50b	50.54a	48.87c	52.94a	53.50b	52.74a
N	5.64b	5.34c	6.33a	5.74a	5.75a	6.51b
S	0.25b	0.25b	0.27a	0.31b	0.30b	0.33a
B	30.30b	29.82b	42.10a	33.04b	33.98b	42.07a
Cu	14.76b	14.71b	19.46a	16.87b	16.44b	20.16a
Fe	67.17a	64.60a	67.32a	82.51a	82.09a	85.22a
Mn	21.20a	19.29a	19.83a	23.95a	20.80b	21.72ab
Mo	4.08b	3.56b	7.68a	5.06b	4.76b	9.77a
Zn	48.39b	44.91b	55.60a	60.54a	56.51a	61.60a
Mold	1.10a	0.00a	0.00a	3.00a	1.10a	0.80a
Purple seed	0.70a	0.00a	0.00a	0.00a	0.00a	0.00a
Phomopsis	8.90a	8.00a	15.70a	1.20a	2.20a	0.30a
Cercospora	3.40a	0.00a	2.30a	0.70a	1.30a	0.30a
Fusarium	1.80a	0.00a	9.00a	0.70a	0.40a	0.70a
*Macrophomena phaseolina*	0.10b	1.30a	0.00b	0.00b	0.00b	0.00b

* *MS breeding lines, Mississippi breeding lines; IL breeding lines, Illinois breeding lines; R8, full maturity stage; within a year, trait, and row, means with the same letter are not significantly different*.

*The experiment was conducted in 2012 and 2013 at the Jamie Whitten Delta States Research Center, Stoneville, MS*.

*Means within a row followed by the same letter are not significantly different at the 5% level as determined by Fishers' LSD test*.

A trait of high interest to producers in a dryland system is the maturity that will maximum yield. A later maturity may have the potential to utilize more sunlight and maximize more of the growing season, but an earlier maturity might better utilize early season rains and avoid late season droughts. The current research provides two different non-irrigated years (environments) to suggest an answer. It is interesting that neither the earliest (MG II, Dwight) nor latest (MG V, AG5606) maturity extreme gave the best return for yield (Table 3). In both years, the highest yielding lines were late IIIs to mid-to-late IVs. In 2012, for the 13 April planting date, the three highest yielding lines matured from 13 August (AG3905, 3797 kg/ha) to 14 August (LG04-1459-6, 3775 kg/ha) to 26 August (DK4866, 4122 kg/ha; Tables 1, 3). For the 30 April planting date in 2013, the three highest yielding lines matured from 24 August (AG3803, 2865 kg/ha and AG3905, 2670 kg/ha) to 7 September (DK4866, 2428 kg/ha; Tables 1, 3). Late MG III AG3905 and mid MG IV DK4866 were included in the highest yielding three lines in each year. In the most stressful year (2013), the late IIIs (AG3803 and AG3905) were the highest yielding. However, in the less stressful year (2012), the IVs (DK4866 and LG04-1459-6) were the highest yielding (Table 3). It might therefore make sense for producers utilizing a dryland production system to plant a strategic mix of both late IIIs and IVs.

## Conclusions

This research demonstrated that two genotypes (25-1-1-4-1-1 and 34-3-1-2-4-1) with ≥80% germinability showed higher seed protein content, although there was no correlation shown between germination and protein across all genotypes. Compared with the checks, seed of two genotypes with ≥80% germinability (25-1-1-4-1-1 and 34-3-1-2-4-1) maintained significantly higher levels of N, P, B, Cu, and Mo, reflecting the possible roles of these nutrients for seed germination and their overall beneficial effects on seed health. The line 25-1-1-4-1-1 will be released as germplasm in 2017 and planned to be given to a public entity by Material Transfer Agreement (MTA); the line 34-3-1-2-4-1 was given to an industry entity by MTA and will be considered for release in the future. These two lines represent the first stage of incorporating high germination traits into cultivars for soybean producers. The correlation between germination and some minerals such as B and Mo in 1 year only may reflect the different responses of nutrients to the growing environment, in our case, drought and high temperature. Seed geminability could partially be affected by hard seed and Ca level, as high geminability genotypes showed lower hard seed and lower Ca content compared to other genotypes. The general low levels of seed infection in most of the genotypes may indicate that these genotypes have some tolerance to these diseases, but more likely it is indicative of dryland growing conditions, where non-irrigated production systems are likely to have fewer seed diseases. In the water stress year (2013), a high germinability genotype (4025-1-1-4-1-1) showed moderately high yield (the fourth highest yielding genotype that year), which may indicate that it has drought stress tolerance. Further, research is needed to select for both high germination and high yield under drought and heat stress. This research will be beneficial to soybean breeders selecting for soybean seed with high seed nutritional values and high germination under dryland conditions.

## Author contributions

NB contributed to the planning, design, analysis, interpretation, and writing. JS contributed to the planning, design, data interpretation, and writing. AM contributed to the analysis, data interpretation, and writing. JR contributed to the planning, design, analysis, data interpretation, and writing. AG contributed to the analysis, data interpretation, writing, revising the manuscript critically with intellectual content.

### Conflict of interest statement

The authors declare that the research was conducted in the absence of any commercial or financial relationships that could be construed as a potential conflict of interest.

## Footnotes

^1^http://www.graphpad.com
